# Treating the most vulnerable: A discursive review of experimental pain in Alzheimer’s disease

**DOI:** 10.1002/nop2.922

**Published:** 2021-06-24

**Authors:** Wm. Larkin Iversen, Ronald L. Cowan, Sebastian Atalla, Sydney S. Englehart, Tanya R. Gure, Karen O. Moss, Claire M. Ryan, Douglas W. Scharre, Kathy D. Wright, Todd B. Monroe

**Affiliations:** ^1^ The Ohio State University College of Nursing The Ohio State University Columbus OH USA; ^2^ Department of Psychiatry The University of Tennessee Health Science Center Memphis TN USA; ^3^ Division of General Internal Medicine and Geriatrics Department of Internal Medicine The Ohio State University Columbus OH USA; ^4^ Vanderbilt Department of Psychiatry and Behavioral Sciences Nashville TN USA; ^5^ Department of Neurology Division of Cognitive Neurology The Ohio State University Wexner Medical Center Columbus OH USA

**Keywords:** dementia, pain, psychophysics

## Abstract

**Aim:**

The purpose of this manuscript is to summarize research on how experimental pain is experienced by adults with Alzheimer's disease (AD) and to translate results into implications for nurses.

**Design:**

This discursive review synthesizes the results of three previous research studies exploring experimental pain in adults with AD.

**Methods:**

Using a series of fictional clinical vignettes, the authors discuss how the results from three previous papers using acute experimental pain can potentially be translated into clinical practice. The authors also introduce the reader to the concept of research‐related psychophysics using introductory definitions and concepts with the impetus to encourage other nurses to consider this research methodology.

**Results:**

Pain characteristics in AD that differ from cognitively intact controls must be explored to properly address pain in this population. Nurses are well positioned to address these issues in order to provide a high quality of care to adults with AD.

## 1 INTRODUCTION

The purpose of this discursive manuscript is to review three distinct studies that used very similar research methods, allowing the results to be critically compared. Following a series of three fictional vignettes describing various clinical scenarios managing pain, we introduce the reader to the research method of pain psychophysics. Next, we discuss how the three research studies described compliment and contrast one another. The discursive review format offers nurses an overview of a research method seldom used by nursing scientists. Psychophysical experiments allow a unique opportunity to examine the neurobiology and psychology of the pain experience in people with dementia.

### 1.1 Fictional vignette #1

Nicholas Delgado is a 67‐year‐old man living in a large Midwestern nursing home. Mr. Delgado was recently diagnosed with mild Alzheimer's disease (AD). Last month, Mr. Delgado visited his neurologist and scored a 15 (range 0–30) on the Mini‐Mental State Exam (MMSE). Last week, Mr. Delgado sustained a slip injury on ice causing a lower back injury. Mr. Delgado's fall was not witnessed. Since the fall, Mr. Delgado has not verbally reported any pain to the nursing staff. The nurse caring for Mr. Delgado has noted that he is showing increased behavioural disturbances since the injury, such as pulling away and acting aggressively during physical examinations. Despite pain being possibly associated with his behavioural disturbances, no comprehensive pain assessment was documented (Ahn & Horgas, 2013; Bruneau, 2014; Buffum et al., 2001). Mr. Delgado's primary care provider (PCP) attributes his behavioural changes to sequelae of dementia, rather than pain, and prescribes psychotropic medications. At a follow‐up visit, Mr. Delgado's behavioural disturbances have not improved. Mr. Delgado's PCP conducts a comprehensive, multidimensional pain assessment and finds that his injury is more severe than originally thought. As such, he is prescribed a common opioid to use as needed. Nursing staff caring for Mr. Delgado note that he is showing decreased behavioural disturbances following this visit.

### 1.2 Fictional vignette #2

Claudia Rowe is a 74‐year‐old woman living in a nursing home, like Mr. Delgado. Ms. Rowe was diagnosed with AD approximately one year ago and scored a 21 on the MMSE last month. Ms. Rowe sustained the same lower back injury as Mr. Delgado after she slipped and fell in the shower last week, which was witnessed by a member of the nursing staff. The nurse caring for Ms. Rowe noted that she shows non‐verbal pain behaviours, such as grimacing, more often during physical examinations than Mr. Delgado; however, Ms. Rowe has not verbally reported significant pain. The nurse has also noted that Ms. Rowe is showing fewer behavioural disturbances than Mr. Delgado. Yesterday, at an appointment with her PCP, Ms. Rowe rated the pain from her back as a 4/10 on a verbal numeric rating scale (NRS). No comprehensive assessment is performed. Ms. Rowe's PCP does not believe this level of pain justifies the use of opioids and prescribes acetaminophen every 4–6 hr. Nursing staff notes that Ms. Rowe continues to show mild behavioural disturbances following this visit.

### 1.3 Fictional vignette #3

Rebecca Hudson is a 71‐year‐old community‐dwelling woman. Ms. Hudson has no diagnosed cognitive impairments and scored a 29 on the MMSE last month during a routine check‐up. Ms. Hudson sustained a lower back injury like Mr. Delgado and Ms. Rowe after slipping and falling while exiting her car. At a visit with her PCP, Ms. Hudson verbally expresses pain during physical examinations and shows strong non‐verbal signs of pain such as grimacing and pulling away. Ms. Hudson rates her pain as a 7/10 on a verbal NRS. Ms. Hudson's PCP prescribes a common opioid to use as needed. Ms. Hudson makes a full recovery from her injury.

The fictional vignettes above describe the challenges nurses face in assessing and managing pain in adults across the spectrum of cognitive dysfunction. Although scientists and clinicians have not yet reached a consensus on the best practices for assessing clinical pain in people with dementia, there is ongoing international research that has thus far resulted in several best practice recommendations (Hadjistavropoulos et al., 2010; Herr et al., 2006, 2019). Several seminal reviews have examined the experimental, or psychophysical, response to pain in adults living with dementia (Defrin et al., 2015; Husebo et al., 2011; Monroe, Gore, et al., 2012). Observational studies of pain in dementia provide important clinical information, but these studies often present inherent limitations that may impact the experience of pain—such as the inability to tightly control the amount of generated pain stimulus—that must be considered when interpreting the results (Monroe, Carter, et al., 2012; Monroe, Carter, et al., 2013; Monroe et al., 2014). Dementia and Alzheimer's disease/AD are used interchangeably throughout the paper.

### 1.4 Background

A historical definition of pain is “whatever the experiencing person says it is, existing whenever he/she says it does” (McCaffery, 1968). This definition presents obvious problems in adults with dementia when there is impaired perception (van Kooten et al., 2016). Persons with dementia are less likely to receive appropriate care for their pain for a myriad of reasons (Scherder et al., 2005). AD is a communicative disorder which may affect one's ability to spontaneously self‐report pain to a care provider, even in the early stages of the disease (Monroe et al., 2014). Clinical pain assessment often relies on verbal reports of pain intensity due to pain's subjective nature; however, these reports may be less reliable in persons with worsening communicative disorders such as AD (Pasero & McCaffery, 2005). Despite the routine administration of pain assessments in clinical settings, documented pain assessment can be greatly improved in persons with dementia residing in long‐term care with written physician orders. Specifically, nurses are more likely to ask about, and residents are more likely to report, pain following written physician orders to assess pain (Monroe et al., 2015). To aid in the assessment of pain in language‐impaired populations, observational tools were developed for the assessment of pain in AD using non‐verbal assessment. These tools require additional training and many necessitate further research to determine their reliability and validity (Herr et al., 2016, 2018, 2019).

Pain research in adults with dementia generally falls into two main categories, clinical pain research and experimental pain research. Each category has their own strengths and weaknesses and both require meticulous attention to research ethics (Monroe, Herr, et al., 2013). Many clinical pain studies examine a population's pain characteristics using measures such as proxy observation or self‐report (Paulson et al., 2014). An advantage of clinical pain studies is that they allow researchers to explore a population's pain characteristics in a naturally occurring setting and do not require the researcher to employ additional painful stimuli. One such commonly studied clinically painful event in older adults is hip fracture. Studies examining the characteristics of hip fractures between cognitively intact and cognitively impaired groups have demonstrated uncertainty about pain treatment in adults with dementia (Jensen‐Dahm et al., 2016) and that persons with dementia receive significantly fewer opioid drug interventions postoperatively (Morrison & Siu, 2000). Studies such as these have provided critical information regarding the problem of pain management in dementia, but clinical pain studies are limited to naturalistic observations and cannot provide information about the response to standardized pain stimuli.

Experimental pain studies offer an alternative for researchers to examine pain in a population with a controlled pain stimulus. In order to understand experimental pain studies, it is first necessary to have an understanding of an additional definition of pain as “An unpleasant sensory and emotional experience associated with, or resembling that associated with, actual or potential tissue damage” (Raja et al., 2020). This definition characterizes pain as a multidimensional sensation divided into multiple components such as sensory, affective, cognitive and behavioural pain (Monroe, Gore, et al., 2012). Experimental pain studies frequently use psychophysical procedures to assess group differences in multiple dimensions of pain—such as sensory and affective pain—since each dimension may have unique neural components (Gracely, 1992; Gracely et al., 1978; Petzke et al., 2003; Treede et al., 1999). Psychophysics are used in pain research to explore how people perceive sensory stimuli (Price et al., 2001). A variety of stimuli have been used to induce pain in psychophysics research in adults with dementia, including mechanical pressure (Cole et al., 2006), electrical shock (Benedetti et al., 2004) and heat or cold (Sommer, 2019).

A variety of experimental procedures exist to test a participant's perception of these stimuli. A common psychophysical procedure in AD is to explore the perception of threshold and tolerance. Threshold is the minimum stimulus needed to experience a specific percept under investigation, such as when an individual first notices pain during stimulus application. Tolerance is the amount of a stimulus which a person recognizes as too painful (Benedetti et al., 1999). An additional application of the threshold procedure that has been successfully used in communicative adults with AD is a perceptually matched paradigm. For example, in a perceptually matched paradigm using mechanical pressure stimulus, the independent variable is a percept such as just noticeable pain, mild pain, or moderate pain while the dependent variable is the pressure at which the percept under investigation is reported (Cole et al., 2006). Individual percepts are then used as a proxy for stimulus intensity, which explores the sensory pain dimension. In order to explore additional dimensions of pain such as affective pain, participants may be asked to rate the unpleasantness associated with each percept (Cole et al., 2006). The perceptually matched paradigm ensures no participant receives a subjective pain rating above the maximum percept used in the study (e.g. moderate pain).

Examining the response to experimental pain provides an opportunity to determine how adults living with dementia experience pain in a controlled set of experiments.

### 1.5 Ethics

Ethical approval was not required as this is a discursive review.

## 2 METHOD

Three studies offer significant insight into different aspects of pain perception in AD (Cowan et al., 2017; Monroe et al., 2016; Romano et al., 2019). The first study, Monroe et al., 2016, is the parent study from which two additional sub‐analyses were conducted. These studies employed a perceptually matched psychophysical heat paradigm modelled after Cole and colleagues’ mechanical pressure study exploring differences in the sensory (measured by intensity) and affective (unpleasantness) pain dimensions between adults with AD and cognitively intact controls (Cole et al., 2006). As this methodology is tailored to each individual participant's perception of the pain percepts, rather than a fixed set of temperatures, it had proven successful in mitigating the risk of any study participant experiencing a perception greater than moderate pain.

## 3 RESULTS

In the parent study, Monroe et al., 2016, all participants were 65 years of age or older and recruited from a large city in the Southern United States. All participants with AD had a formal diagnosis and were verbally communicative. Cognition was assessed using the Folstein Mini‐Mental State Examination (MMSE) (Folstein et al., 1975). This parent study included 40 cognitively intact controls with a median MMSE of 30, and 40 participants with AD who had a median MMSE of 19.5. The groups were balanced by age and sex (Monroe et al., 2016). All participants were told to not use pain medications within 24 hr of psychophysical testing and detailed inclusion and exclusion criteria are previously published (Monroe et al., 2016).

Cowan et al., 2017 used a subsample of the parent study to specifically explore sex differences in response to the psychophysical assessment in participants screening positive for dementia as defined by a MMSE score of 23 or less (Folstein et al., 1975). The rationale for the MMSE cut‐off of 23 was that the authors sought to analyse subjects meeting the definition for “likely dementia.” In the parent study, some of the AD participants with MMSE scores between 23 and 26 may have actually been better categorized as having mild cognitive impairment (MCI) (Folstein et al., 1975). This approach resulted in 14 males and 14 females diagnosed with AD with an MMSE score of 23 or less. This study resulted in a sample of females with a median MMSE of 15.5 while males had a median MMSE of 16. The female AD group displayed greater current (pain right now) and average pain scores than the male AD group as measured by the Brief Pain Inventory Short Form (Cleeland & Ryan, 1994; Tan et al., 2004).

In the third study, the authors aimed to examine sex differences in response to psychophysical assessment across the cognitive spectrum, including both cognitively intact controls and participants with MCI and AD in each group (Romano et al., 2019). The authors excluded participants with MMSE scores below 10 to account for the potential confound of adults with more severe AD since the parent study included communicative adults with AD who scored less than 10 on the MMSE. This analysis included 38 females (Control = 14; MCI = 8; AD = 16) and 38 males (Control = 17; MCI = 6; AD = 15). Across all levels of cognition, the female group had a median MMSE of 27 and the male group had a median MMSE of 28 (Romano et al., 2019).

## 4 DISCUSSION

### 4.1 Discussion of sensory pain findings (intensity)

Findings from the parent study demonstrated that relative to cognitively intact controls, participants with AD required higher temperatures to report the perception of warmth, mild pain and moderate pain (all *p* < .05) (Monroe et al., 2016). There is little scientific consensus on sensory pain perception in AD (Defrin et al., 2015). These findings are partially supported by results from other research groups using various stimuli and compound evidence that sensory pain thresholds may be higher in persons affected by AD (Cole et al., 2006). Importantly, these results provide evidence that persons with AD may require more time to report perceptually matched painful stimuli relative to cognitively intact controls. It should be noted that the perceptually matched psychophysical paradigm used in the study does require specific training for participants to ensure understanding and requires persons to recognize each percept, an ability which may be altered in cognitively impaired individuals (Cole et al., 2006). For example, it is recognized that pain is both under‐detected and under‐reported in adults with dementia (McAuliffe et al., 2009).

When examining the association of MMSE scores with temperature sensitivity, the AD group demonstrated that lower MMSE scores required higher temperatures to report warmth only, with no significant differences between mild pain or moderate pain (*p* < .05) (Monroe et al., 2016). These findings suggest that worsening cognition may increase the amount of sensory stimulation required for a person with AD to detect innocuous stimuli but not painful stimuli. This finding indicates that AD pathology impacts mild and moderate pain perception irrespective of the degree of cognitive impairment. Benedetti et al. (1999) found a significant inverse correlation between MMSE scores and pain tolerance in persons with AD, with more cognitively impaired persons having higher pain tolerances. This study did not find threshold differences between the two groups. This study included a mixture of electrical stimulation and ischaemia rather than heat pain. In contrast, Monroe et al. examined the perception of warmth, which may make the studies not directly comparable because the different modalities (e.g. thermal versus electric shock) may have different biological mechanisms (e.g. receptors, fibres) affecting the perception of pain (Basbaum et al., 2009; Benedetti et al., 1999; Monroe et al., 2016; Scherder et al., 2005).

The second study found that when examining sex differences in persons with MMSE scores of 23 or less, women reported the percepts of mild pain (*Cohen's* *d* = 0.72, *p* = .051) and moderate pain (*Cohen's* *d* = 0.80, *p* = .036) at lower temperatures than men (Cowan et al., 2017). These particular findings are supported by a large body of literature demonstrating that cognitively intact adult females tend to be more sensitive to pain when compared to cognitively intact males (Fillingim et al., 2009). The similarity of these findings may provide evidence that the sexual dimorphism of sensory pain processing is still partially intact in AD and suggests that broader aspects of sensory pain processing may still be intact in AD as well.

The third study examined sex differences in pain perception across the cognitive spectrum and found that women required less heat to report the percepts of mild pain and moderate pain (all *p* < .05), which mirrors the results of the second study in AD only and much of the current literature in cognitively intact controls (Cowan et al., 2017; Fillingim et al., 2009; Romano et al., 2019). These findings suggest that the sexual dimorphism of sensory pain perception may remain intact regardless of the degree of cognitive impairment.

Moreover, the third study revealed an inverse relationship between MMSE scores and the temperature required to report warmth in both males and females (*p* < .05), similar to what the parent study revealed in AD only (Romano et al., 2019). This finding suggests that worsening global cognition increases the temperature required to report warmth irrespective of AD diagnosis. Furthermore, due to the similarity of these findings relative to AD only, it is plausible that the neural mechanism leading to this effect presents early and remains persistent through the course of cognitive decline. Interestingly, Pickering et al. reported no significant correlation of MMSE scores with heat pain thresholds or tolerance in cognitively intact controls (Pickering et al., 2002). This finding tends to support previous results that AD pathology impacts pain perception. Additionally, the cognitive demands of the perceptually matched psychophysical paradigm may play a role in these findings (Defrin et al., 2015). Replication of these findings using less cognitively demanding psychophysical paradigms will generate a better understanding of the relationship between global cognition and sensory pain perception.

### 4.2 Discussion of affective pain findings (unpleasantness)

While the parent study found that participants with AD required higher temperatures to report the sensory pain percepts of warmth, mild pain and moderate pain relative to cognitively intact controls, it did not find between‐groups differences in unpleasantness ratings for any of these percepts (Monroe et al., 2016). While there is no current consensus on affective pain differences between cognitively intact controls and persons with AD, Cole et al., 2006 reported higher unpleasantness ratings for their AD group along with no reduction in brain activation of regions believed to be responsible for affective pain processing (Cole et al., 2006). These results suggest that persons with AD may report the same pain as less intense than cognitively intact controls from a sensory pain perspective but may have similar emotional responses to the same pain from an affective pain perspective.

The second study found that males with AD reported higher unpleasantness ratings for mild pain (*Cohen's* *d* = 0.82, *p* = .072) and moderate pain (*Cohen's d = *1.32, *p* = .006) relative to females with AD. Effect sizes demonstrated notable differences on the impact of pain unpleasantness by sex (Cowan et al., 2017). Interestingly, this finding differs from the literature in cognitively intact adults, with studies typically finding that females report higher unpleasantness ratings relative to males (Fillingim et al., 2009). Unfortunately, there is very little research exploring sex differences in experimental pain in adults with AD. However, the paradoxical nature of these findings suggests inherent sexual dimorphism in the impact of AD on affective pain states. Previous neuroimaging work has reported sexually dimorphic grey matter atrophy of pain‐related brain areas, such as the thalamus and insula, in AD (Skup et al., 2011). Therefore, it is plausible that these sex differences in atrophy would produce sex differences in pain that would differ between cognitively intact controls and adults with AD, due to direct alterations of pain processing or downstream effects on higher level networks associated with pain responses (Monroe et al., 2018).

Finally, the third study found that males across the cognitive spectrum consistently report higher unpleasantness ratings for moderate pain relative to females (*p* < .05) (Romano et al., 2019). These findings mirror the previous results exploring sex differences in pain unpleasantness ratings in AD only (Cowan et al., 2017). As previously mentioned, these findings diverge from the literature exploring pain unpleasantness ratings in cognitively intact adults (Fillingim et al., 2009). There are limitations of perceptually matched psychophysical paradigms, and these initial studies on sex differences are small pilot studies. The findings need to be replicated in larger investigations, but these sex differences in sensory and affective pain may partially explain the lack of consensus in the literature regarding experimental pain in AD.

## 5 CONCLUSIONS

While there is no current consensus on how persons with AD perceive pain, the general consensus is that adults with AD experience pain in a similar way to cognitively intact controls. This notion is highlighted by the preservation of some sexually dimorphic sensory pain characteristics in AD. This implies that care providers must be aware of sexual differences in sensory pain perception in all patients in order to deliver the highest quality of care possible.

However, the sexual dimorphism of affective pain in adults with AD may differ from cognitively intact controls, namely that males with AD reported higher unpleasantness than females with AD. If these findings hold true, it will be important for care providers to receive adequate education on these paradoxical differences in order to deliver the highest quality of care possible. This interaction effect between sex and AD status on affective pain ratings provides further evidence that the call to explore sex differences in AD must be answered (Mazure & Swendsen, 2016). Further exploration of pain‐network differences between male and female brains, sexual dimorphism in neurodegeneration (Skup et al., 2011) and the potential implication of sex chromosomes in AD (Bajic et al., 2020) will help care providers tailor treatment to their patients.

Unfortunately, because clinical pain assessment often involves sensory pain only, persons with AD are at risk for a lack of recognition of their affective/emotional pain. Additionally, the discrepancy between sensory and affective pain reports may contribute to healthcare providers being less certain about assessing pain in persons with AD. This uncertainty may further predispose persons with AD, particularly in long‐term care, to delayed or to not be given pharmacologic and non‐pharmacological pain interventions (Bruneau, 2014; Gilmore‐Bykovskyi & Bowers, 2013). To further complicate the matter, pain and behavioural disturbance, such as agitation, may be significantly correlated in persons with dementia (Ahn & Horgas, 2013; Bruneau, 2014; Buffum et al., 2001). These behavioural disturbances therefore may be improperly attributed to the behavioural and psychological symptoms of dementia rather than pain, which leads to the improper emphasis of prescribing psychotropics over analgesics (Kovach et al., 2000). This is where the nurse's assessment is critical to the proper identification and subsequent management of pain in these individuals. Unfortunately, on top of delays to proper pain treatment, the inappropriate use of psychotropics in dementia may have adverse effects in and of itself (Bruneau, 2014). Moreover, behavioural disturbances stemming from unrecognized pain can further burden caregivers and lead to institutionalization (Desai & Grossberg, 2001). Altogether, this lack of recognition may lead to increased suffering and diminished quality of life for those living with AD. This potential for decreased quality of life, in addition to the potential sex differences in pain perception in AD, underscores the need for care providers to employ standardized and tailored assessments for recognizing pain in patients with AD, rather than relying on intuition or assessment techniques that may not be valid in this population.

Experimental pain assessment is a growing field, and future innovations will help to achieve a better understanding of pain in AD. Threshold‐based psychophysical procedures, such as the perceptually matched paradigm, are ideal modalities for assessment because the pain stimulus accounts for individual differences in perception. Additionally, the perceptually matched paradigm allows the participant to have control over the amount of generated stimulus. However, threshold‐based psychophysical procedures may be more reliant on cognitive ability, and this limitation must be considered in individuals with severe cognitive impairment. The use of simpler psychophysical paradigms may lead to more reliable pain assessment in AD. One such paradigm is a fixed temperature paradigm. In a fixed temperature paradigm, each participant is administered the same temperature and asked to rate its intensity and unpleasantness, rather than being asked to stop the stimulus at a designated percept such as mild pain. Another strength of using fixed temperatures is the elimination of individual variations in temperatures to achieve identical perceptions. However, researchers must carefully select the temperatures used in order to ensure that a participant's subjective interpretation of the temperatures is never extremely painful.

Finally, further integration of valid and reliable measures in this field will allow for more objective evidence of the pain experience. For example, Kunz et al., 2007 found that persons with dementia show an increased frequency and intensity of facial pain responses during pressure pain (Kunz et al., 2007), which has been supported by work in other laboratories (Beach et al., 2016). Furthermore, they did not find any self‐report intensity differences between cognitively intact controls and participants with AD. While this study provided important knowledge, it did not delineate between sensory and affective facial pain responses. However, later work by Kunz et al. (2012) found that the sensory and affective pain dimensions may produce unique facial responses (Kunz et al., 2012). Further validation of these findings and their combination with biomarkers, such as neuroimaging, may guide more effective experimental paradigms for the assessment of pain in persons with dementia.

### 5.1 Relevance to clinical practice

Nurses among other healthcare providers face numerous challenges in assessing pain in persons with AD, particularly as it progresses and verbal communication lessens. To address this challenge, healthcare providers should implement best practice guidelines and recommendations for pain assessment in AD, such as the use of multidimensional and non‐verbal pain assessment. Additionally, false beliefs surrounding pain perception and treatment in AD should be corrected through proper education and training.

Based on this review and other works in the field (Gloth, 2011; Herr et al., 2019), we recommend the following practice guidelines for nurses and all clinicians/healthcare providers when assessing and managing pain in adults with AD and other forms of dementia.

1. Use multidimensional and non‐verbal pain assessment techniques.
2. Ensure comprehensive pain assessments are standard practice for all patient visits.
3. Mandatory education and training to improve the knowledge and comfort of healthcare providers who routinely care for patients with dementia in pain assessment and management.
4. Develop novel ways to uniformly include caregivers and/or proxies in implementation of a care plan to improve pain identification and management in this population.
5. Consider using scheduled, low‐dose analgesics rather than *pro re nata* or as needed.

## CONFLICT OF INTEREST

The authors have no conflicts of interest to report.

## AUTHOR CONTRIBUTIONS

All authors qualify for authorship based on the criteria set forth by *Nursing Open*.

## Data Availability

As this is a review, no new data were collected or analysed for this manuscript.
